# The prognostic impact of subclonal IDH1 mutation in grade 2–4 astrocytomas

**DOI:** 10.1093/noajnl/vdad069

**Published:** 2023-05-29

**Authors:** Meenakshi Vij, Raquel T Yokoda, Omid Rashidipour, Ivy Tran, Varshini Vasudevaraja, Matija Snuderl, Raymund L Yong, William S Cobb, Melissa Umphlett, Jamie M Walker, Nadejda M Tsankova, Timothy E Richardson

**Affiliations:** Department of Pathology, Molecular and Cell-Based Medicine, Icahn School of Medicine at Mount Sinai, New York, New York, USA; Department of Pathology, Molecular and Cell-Based Medicine, Icahn School of Medicine at Mount Sinai, New York, New York, USA; Department of Pathology, Molecular and Cell-Based Medicine, Icahn School of Medicine at Mount Sinai, New York, New York, USA; Department of Pathology, NYU Langone Health, New York, New York, USA; Department of Pathology, NYU Langone Health, New York, New York, USA; Department of Pathology, NYU Langone Health, New York, New York, USA; Department of Neurosurgery, Icahn School of Medicine at Mount Sinai, New York, New York, USA; The Valley Hospital, Ridgewood, New Jersey, USA; Department of Pathology, Molecular and Cell-Based Medicine, Icahn School of Medicine at Mount Sinai, New York, New York, USA; Department of Pathology, Molecular and Cell-Based Medicine, Icahn School of Medicine at Mount Sinai, New York, New York, USA; Nash Family Department of Neuroscience, Icahn School of Medicine at Mount Sinai, New York, New York, USA; Department of Pathology, Molecular and Cell-Based Medicine, Icahn School of Medicine at Mount Sinai, New York, New York, USA; Nash Family Department of Neuroscience, Icahn School of Medicine at Mount Sinai, New York, New York, USA; Department of Pathology, Molecular and Cell-Based Medicine, Icahn School of Medicine at Mount Sinai, New York, New York, USA

**Keywords:** astrocytoma, glioblastoma, IDH mutation, mosaic IDH1 R132H immunohistochemistry, oligodendroglioma, subclonal mutation

## Abstract

**Background:**

Isocitrate dehydrogenase (IDH) mutations are thought to represent an early oncogenic event in glioma evolution, found with high penetrance across tumor cells; however, in rare cases, IDH mutation may exist only in a small subset of the total tumor cells (subclonal IDH mutation).

**Methods:**

We present 2 institutional cases with subclonal *IDH1* R132H mutation. In addition, 2 large publicly available cohorts of IDH-mutant astrocytomas were mined for cases harboring subclonal IDH mutations (defined as tumor cell fraction with IDH mutation ≤0.67) and the clinical and molecular features of these subclonal cases were compared to clonal IDH-mutant astrocytomas.

**Results:**

Immunohistochemistry (IHC) performed on 2 institutional World Health Organization grade 4 IDH-mutant astrocytomas revealed only a minority of tumor cells in each case with IDH1 R132H mutant protein, and next-generation sequencing (NGS) revealed remarkably low *IDH1* variant allele frequencies compared to other pathogenic mutations, including *TP53* and/or *ATRX*. DNA methylation classified the first tumor as high-grade IDH-mutant astrocytoma with high confidence (0.98 scores). In the publicly available datasets, subclonal IDH mutation was present in 3.9% of IDH-mutant astrocytomas (18/466 tumors). Compared to clonal IDH-mutant astrocytomas *(n* = 156), subclonal cases demonstrated worse overall survival in grades 3 (*P* = .0106) and 4 (*P* = .0184).

**Conclusions:**

While rare, subclonal *IDH1* mutations are present in a subset of IDH-mutant astrocytomas of all grades, which may lead to a mismatch between IHC results and genetic/epigenetic classification. These findings suggest a possible prognostic role of IDH mutation subclonality, and highlight the potential clinical utility of quantitative *IDH1* mutation evaluation by IHC and NGS.

**Key Points**

- Subclonal IDH mutation is a rare finding in IDH-mutant astrocytoma cohorts, but can be detected by routine immunohistochemical and next-generation sequencing studies.
- Subclonal IDH mutation may have an adverse effect on survival in patients with grades 3–4 IDH-mutant astrocytoma.

**Importance of the Study**

IDH-mutant astrocytomas are biologically distinct from IDH-wild-type glioblastoma and have significantly better progression/recurrence-free and overall survival after initial resection. However, there remains significant heterogeneity of clinical outcomes in IDH-mutant astrocytomas that is unexplained by the current glioma classification and grading system alone. Our study demonstrates that a small subset of IDH-mutant astrocytomas has a low fraction of tumor cells with IDH mutations, and these subclonal IDH-mutant astrocytomas have shorter overall survival intervals compared to grade-matched IDH-mutant astrocytomas with high IDH mutation tumor cell fraction (TCF)s. These findings suggest a potentially overlooked source of variability in clinical outcome amongst IDH-mutant astrocytomas, and demonstrate the importance of quantitative IDH mutation evaluation by next-generation sequencing.

Diffusely infiltrating glial neoplasms are among the most common intracranial tumors, second only to meningioma. Historically, these tumors have been defined based on histologic features alone. However, in the past decade, the discovery of numerous molecular features which could be used to subdivide diffuse gliomas into improved prognostic groups has led to a revolution in the definition and grading of these tumors. Currently, adult-type diffusely infiltrating gliomas are subdivided into IDH-mutant astrocytoma, IDH-mutant and 1p/19q-codeleted oligodendroglioma, and IDH-wild-type glioblastoma.^1^ With an incidence of 0.44 per 100 000 individuals and nearly 3000 cases annually in the United States, IDH-mutant astrocytoma comprises approximately 11% of all of these diffusely infiltrating gliomas.^2^

Since the prognostic importance of *IDH1/2* mutations was demonstrated in astrocytic neoplasms,^3–5^ much work has been done to further refine this category and to identify additional prognostic factors to explain the remaining heterogeneity in clinical outcomes in these tumors.^6–11^ This led to the inclusion of homozygous *CDKN2A/B* deletion as a codified molecular criterion for grade 4 status in IDH-mutant astrocytoma, equivalent to the histologic findings of microvascular proliferation and palisading necrosis.^1,12^ Numerous other studies have suggested additional prognostic factors including *CDK4* amplification,^8,9,11,13^ mismatch-repair deficits,^14^ and genome-wide copy number variation (CNV)/chromosomal instability (CIN).^10,15–18^ One additional factor that may have significant prognostic implications in gliomas is the presence of subclonal populations of tumor cells with mosaic expression of certain molecular alterations, including IDH mutations.^19–27^

In this report, we discuss 2 institutional cases of WHO grade 4 IDH-mutant astrocytoma with mosaic IDH1 R132H expression by immunohistochemistry and very low *IDH1* variant allele frequencies (VAF) compared to other identified pathogenic mutations, including *TP53* and/or *ATRX*, corresponding to subclonal *IDH1* mutation (ie, a low *IDH1* mutation TCF). We also evaluate 2 large, independent, publicly available datasets to further investigate the frequency and clinical importance of subclonal IDH mutation.

## Methods

### Institutional Cases, Histology, and Immunohistochemistry

Two astrocytomas with subclonal *IDH1* R132H mutation were identified out of 108 institutional IDH-mutant gliomas, queried between January 1, 2019 and March 1, 2023 (1.9%). All tissue samples were obtained under approved Icahn School of Medicine at Mount Sinai Institutional Review Board protocols and appropriate consent. Hematoxylin and eosin (H&E)-stained slides were prepared on 4 µm-thick sections of formalin-fixed paraffin-embedded tissue using standard protocols. Immunohistochemistry (IHC) was performed on 4 µm-thick sections after heat-induced epitope retrieval using CC1 (Ventana, Tucson, AZ, USA). IHC panels performed on both included institutional cases consisted of: GFAP (pre-dilute (1:1 dilution); GA52461-2; Agilent, Santa Clara, CA, USA), OLIG2 (1:100 dilution; ZMS1019; Sigma-Aldrich, St. Louis, MO, USA), IDH1 R132H (1:50 dilution; DIA-H09; Dianova, Hamburg, Germany), ATRX (1:200 dilution; MABE1789; Sigma-Aldrich), p53 (pre-dilute; GA61661-2; Agilent), Ki-67/MIB-1 (pre-dilute; GA62661-2; Agilent), MSH2 (pre-dilute; GA08561-2; Agilent), MSH6 (pre-dilute; GA08661-2; Agilent), PMS2 (pre-dilute; GA08761-2; Agilent), and MLH1 (pre-dilute; GA07961-2; Agilent) using Dako Omnis or Ventana UltraView Universal DAB detection kits.

### Molecular Profiling

Clinically validated next-generation DNA sequencing (NGS) was performed on institutional cases at FoundationOne Laboratories (https://www.foundationmedicine.com/test/foundationone-cdx) (Foundation Medicine, Inc., Cambridge, MA) using targeted high throughput hybridization-based capture technology for detection of genomic alterations. For institutional case 1, whole genome DNA methylation profiling and classification was performed at the New York University Molecular Pathology laboratory using the Illumina EPIC Human Methylation array, which assesses 850 000 CpG sites, according to the manufacturer's protocol and is used for tumor classification (www.molecularneuropathology.org), as previously described.^15,28–32^

### Public Dataset Analysis

To identify similar cases in public datasets, we queried the cBioPortal For Cancer Genomics (http://www.cbioportal.org/)^33,34^ to evaluate 2 large cohorts of diffuse gliomas totaling 2126^7,35^ cases. The original histologic diagnoses reported included “diffuse glioma,” “oligodendroglioma,” “anaplastic oligodendroglioma,” “oligoastrocytoma,” “anaplastic oligoastrocytoma,” “astrocytoma,” “anaplastic astrocytoma,” and “glioblastoma.” All cases represent the first known biopsy or resection specimen and were manually reclassified using *IDH1/2*, *ATRX*, *TP53*, and 1p/19q status according to the 2021 World Health Organization (WHO) Classification of Central Nervous System Tumors, leaving a combined total of 466 IDH-mutant astrocytomas, WHO grades 2-4.^1^ Mutation, global and individual CNV, gene expression data, methylation profile status, tumor purity, and survival data were downloaded and analyzed as previously described in detail.^13,17,18,36,37^ The variant allele frequency (VAF) for each pathogenic mutation was defined as the ratio of mutant alleles divided by total alleles and was corrected in instances of homozygous mutation or allelic loss in the case of *TP53*, as well as gender in the case of *ATRX*.^38^ The TCF with IDH mutation was estimated using the ratio of *IDH1* or *IDH2* VAF divided by the corrected VAF of other pathogenic mutations that were found at clonal levels^23^ (*TP53* or *ATRX* if *TP53* mutation was not present), and a conservative threshold of ≤0.67 was used as a working definition of subclonal IDH mutation. Eighteen cases were identified that met these criteria for subclonal IDH-mutant astrocytoma, and were compared to 156 cases with clonal IDH mutation (IDH TCF ≥ *TP53* and/or *ATRX*). This simplified method of determining the TCF with *IDH1* mutation had an 85% concordance rate for identifying subclonal mutations and a 95% concordance rate for identifying clonal mutations compared to previously proposed methods for determining mutation clonality.^24,27^

### Statistical Analysis

The significance of differences between Kaplan–Meier survival curves were calculated using the Mantel-Cox test (Log-rank test). Differences in patient age and CNV were evaluated using student’s *t*-test. Proportion of cases with specific molecular alterations as well as histologic grade and patient gender were calculated using Fisher’s Exact test. All statistical calculations were performed with GraphPad Prism version 9 (GraphPad, La Jolla, CA).

## Results-

### Institutional Case Histories

#### Institutional case 1:.

**Figure 1.**
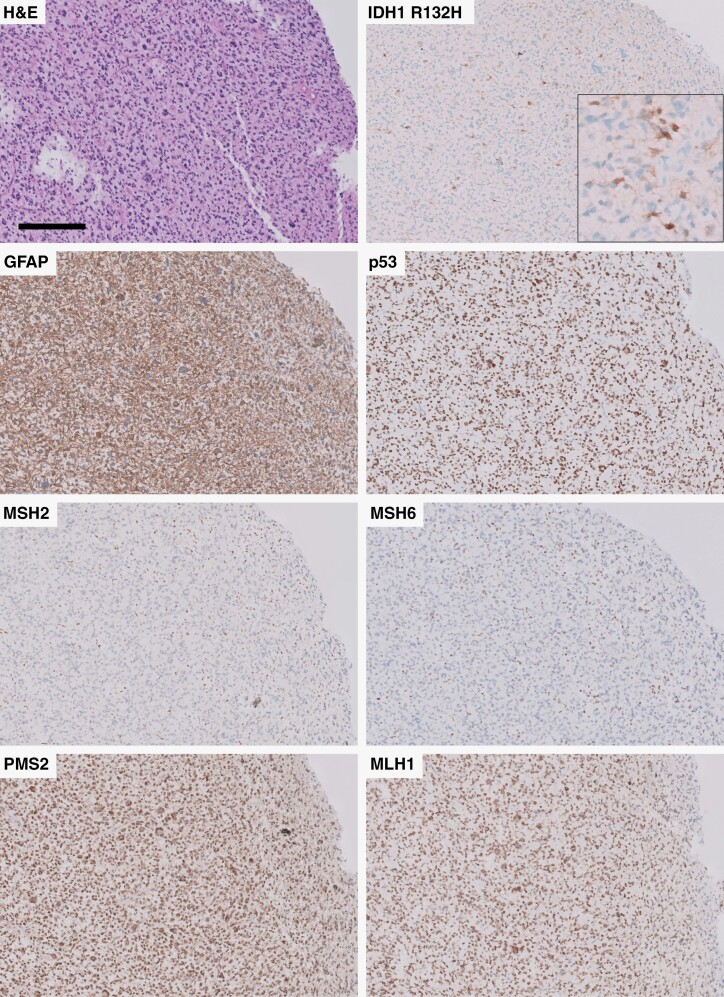
Immunohistochemical findings in institutional case 1, demonstrating mosaic IDH1 R132H staining and loss of nuclear MSH2 and MSH6 expression. All panels were taken at 100x except insert in panel B, which was taken at 400x. Scale bar = 200 µm and applies to each panel.

**Figure 2.**
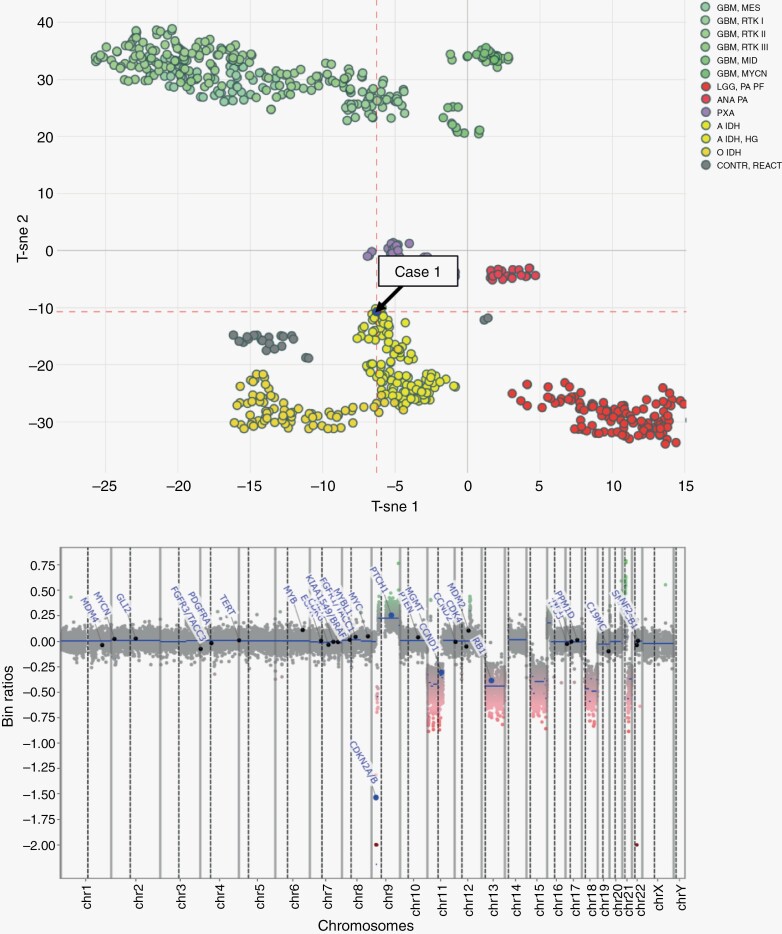
T-distributed stochastic neighbor embedding (T-SNE) plot and copy number profile plot for institutional case 1, demonstrating epigenetic features consistent with IDH-mutant astrocytoma, high grade.

A 68-year-old female with a 5.3 cm complex solid-cystic necrotic mass in the right frontal lobe convexity and an adjacent 5.0 cm complex, predominantly cystic mass in the right parasylvian cortex. The resected tissue demonstrated brisk mitotic activity, microvascular proliferation, and palisading necrosis. Immunohistochemical (IHC) studies demonstrated strong GFAP and OLIG2 staining, approximately 50% MIB-1/Ki-67 proliferation index, frequent nuclear p53 staining, loss of p16 staining, intact ATRX expression, and largely negative IDH1 R132H expression, although scattered cells demonstrated positive cytoplasmic expression (<1% of total tumor cells) (Figure 1). In addition, there was partial loss of MSH2 and MSH6 with intact nuclear expression for MLH1 and PMS2. NGS results demonstrated low *IDH1* R132H variant allele frequency (VAF) of 0.8% with significantly higher VAF for additional mutations, including *TP53* (89% VAF) and *PIK3R1* (48% VAF), and homozygous *CDKN2A/B* deletion, among other alterations (Table 1). In addition, in 87% of cells, there was an *MSH2* mutation and a high overall tumor mutation burden (14 mutations/Mb). No additional alterations were suggestive of IDH-wild-type glioblastoma (*EGFR* amplification, *TERT* promoter mutation, or simultaneous gain of chromosome 7/loss of chromosome 10) were detected. Despite the extremely low *IDH1* VAF,^39^ DNA methylation profiling was consistent with high-grade IDH-mutant astrocytoma with a high degree of certainty (confidence score = 0.98) (Figure 2).

**Table 1.** Clinical and Molecular Features in Institutional Astrocytomas With Subclonal IDH Mutations

Case	Age	Gender	Imaging	2021 WHO Diagnosis	Molecular Features	Methylation Profiling Results
1	68	F	5.3 cm complex solid-cystic right frontal lobe mass	IDH-mutant astrocytoma, Grade 4	**SNV:** *IDH1, TP53, PIK3R1, PIK3CA, BCOR, ALK, MSH2;* **Loss:** *MTAP, CDKN2A/B*; *MGMT* promoter methylation; 14 mutations/Mb	IDH-mutant astrocytoma, high-grade (0.98 confidence score)
2	42	F	3.8 cm left frontotemporal-insular mass with heterogeneous enhancement	IDH-mutant astrocytoma, Grade 4	**SNV:** *IDH1, TP53,* ATRX; **Loss:***MTAP, CDKN2A/B*; *MGMT* promoter methylation; 6 mutations/Mb	Not performed

SNV, single nucleotide variant.

#### *Institutional case 2*:.

**Figure 3.**
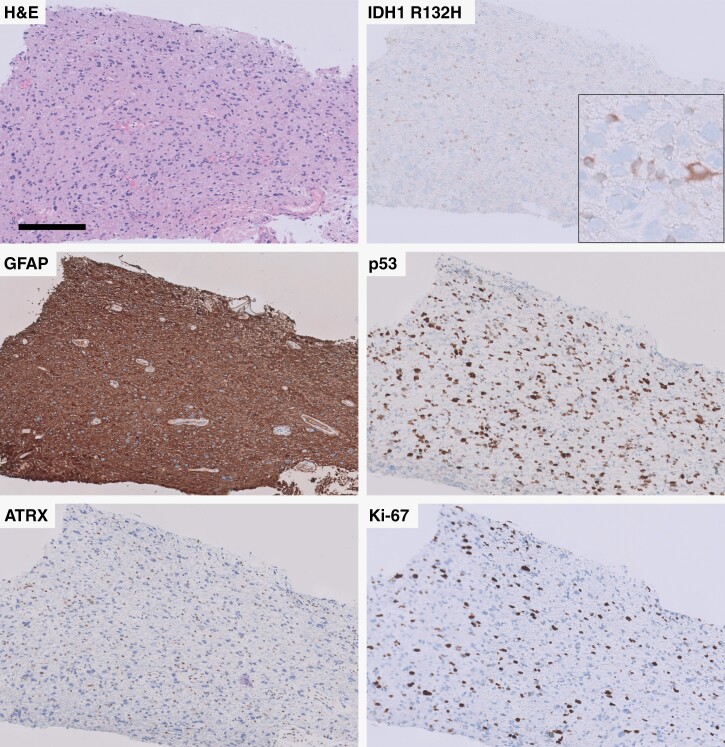
Immunohistochemical findings in institutional case 2, demonstrating mosaic IDH1 R132H in relation to p53 and ATRX staining. All panels were taken at 100x except insert in panel B, which was taken at 400x. Scale bar = 200 µm and applies to each panel.

A 42-year-old female with a past medical history of seizures and left fronto-insular glioma, not otherwise specified that was originally biopsied in 2012 and subsequently treated with radiotherapy and temozolomide, developed memory difficulties and right upper and lower extremity weakness in 2022. MRI demonstrated enhancing progression of her left frontotemporal-insular diffusely infiltrating glioma. Subsequent biopsy demonstrated a hypercellular infiltrating glioma with mitotic figures, insipient microvascular proliferation, and necrosis most consistent with treatment effect. IHC studies demonstrated GFAP and OLIG2 immunoreactivity, an 18% MIB-1/Ki-67 proliferation index, increased p53 staining, loss of p16 staining, loss of nuclear ATRX expression, and scattered IDH1 R132H reactivity (Figure 3). NGS demonstrated an *IDH1* R132H mutation (11% VAF), *ATRX* mutation (22% VAF), *TP53* mutation (69% VAF), and homozygous *CDKN2A/B* loss (Table 1). No tissue was available from the 2012 biopsy specimen for additional molecular testing.

#### Frequency and effect of subclonal IDH mutation

To further investigate the long-term impact of low IDH mutation VAF, we identified 18 subclonal IDH-mutant astrocytoma cases (3.9% of total IDH-mutant astrocytomas) in publicly available datasets^7,35^ with IDH-mutant TCF ≤0.67 and an additional 156 cases with clonal *IDH1* mutation. TCF was established as the ratio between the *IDH1* mutation VAF and the VAF of additional clonal pathogenic mutations (*TP53* and/or *ATRX*) after correction for heterozygous, homozygous, and hemizygous mutations, as this information was uniformly available in all institutional and publicly available cases, and allows for simple identification of cases with low IDH-mutant TCF (subclonal IDH-mutant cell population) using only the results from standard NGS reports. Subclonal IDH mutation appears to occur across all WHO grades, and no significant difference was identified in the gender, age, grade, O-6-methylguanine-DNA methyltransferase (MGMT) promoter methylation frequency, or level of overall CNV between subclonal and clonal IDH-mutant astrocytoma cohorts or in grade-for-grade analysis (Table 2). One of the institutional cases had mutations in a mismatch-repair gene (*MSH2*); however, only 1 of the 18 subclonal IDH-mutant cases had such a mutation (*PMS2*), which was not significantly higher than the clonal IDH-mutant cohort. Global DNA methylation profiling and classification were available for 13 of the 18 subclonal IDH-mutant astrocytomas. All 13 of these matched with IDH-mutant, non-1p/19q-codeleted astrocytoma; 3 of these were consistent with glioma CpG island methylation phenotype (G-CIMP) “low” and the remaining 10 were G-CIMP “high.”^40,41^

**Table 2.** Clinical and Molecular Features in Astrocytomas With and Without Subclonal IDH Mutations

Group	*n*	Gender (M:F)	Age (Years)	2021 WHO Grade (2:3:4)	Homozygous *CDKN2A* Deletion	*MGMT* Promoter Methylation*	MMR Gene Mutation	CNV (%)	Median PFS (Months)	Median OS (Months)
2021 WHO Grade 2										
Subclonal IDH	6	6:0	31.8 ± 4.9	N/A	N/A	6 (100%)	0 (0%)	6.6 ± 0.6	180.0	196.0
Clonal IDH	64	40:24	37.3 ± 1.7	N/A	N/A	40 (75.4%)	2 (3.1%)	7.5 ± 1.0	95.1	144.9
*P*-value	-	0.0874	0.3430	-	-	0.3220	>0.9999	0.7854	0.1355	0.3668
2021 WHO Grade 3										
Subclonal IDH	8	4:4	37.6 ± 4.5	N/A	N/A	7 (100%)	0 (0%)	26.7 ± 7.5	38.0	50.8
Clonal IDH	54	30:24	36.2 ± 0.8	N/A	N/A	33 (73.3%)	1 (1.9%)	15.9 ± 2.1	114.0	114.0
*P*-value	-	>0.9999	0.6011	-	-	0.1808	>0.9999	0.0838	0.0955	**0.0106**
2021 WHO Grade 4										
Subclonal IDH	4	2:2	32.8 ± 1.2	N/A	3 (75%)	2 (66.7%)	1 (25%)	24.8 ± 5.8	17.4	32.4
Clonal IDH	38	20:18	36.5 ± 1.8	N/A	20 (52.6%)	23 (79.3%)	3 (7.9%)	23.1 ± 2.2	36.0	57.1
*P*-value	-	>0.9999	0.5141	-	0.6135	0.5363	0.3405	0.8109	0.1725	**0.0184**
Combined										
Subclonal IDH	18	12:6	34.6 ± 2.3	64:54:38	3 (16.7%)	15 (93.8%)	1 (5.6%)	18.7 ± 4.1	38.0	50.8
Clonal IDH	156	90:66	36.8 ± 1.0	6:8:4	20 (12.8%)	96 (75.6%)	6 (3.8%)	16.2 ± 1.1	65.4	114.0
*P*-value	-	0.6148	0.4715	0.7012	0.7114	0.1217	0.5410	0.4786	0.4712	**0.0033**

MMR, mismatch repair; CNV, copy number variation; PFS, progression-free survival; OS, overall survival; bold indicates statistical significance to a level of <0.05; *not all cases had available *MGMT* promoter methylation data.

**Figure 4.**
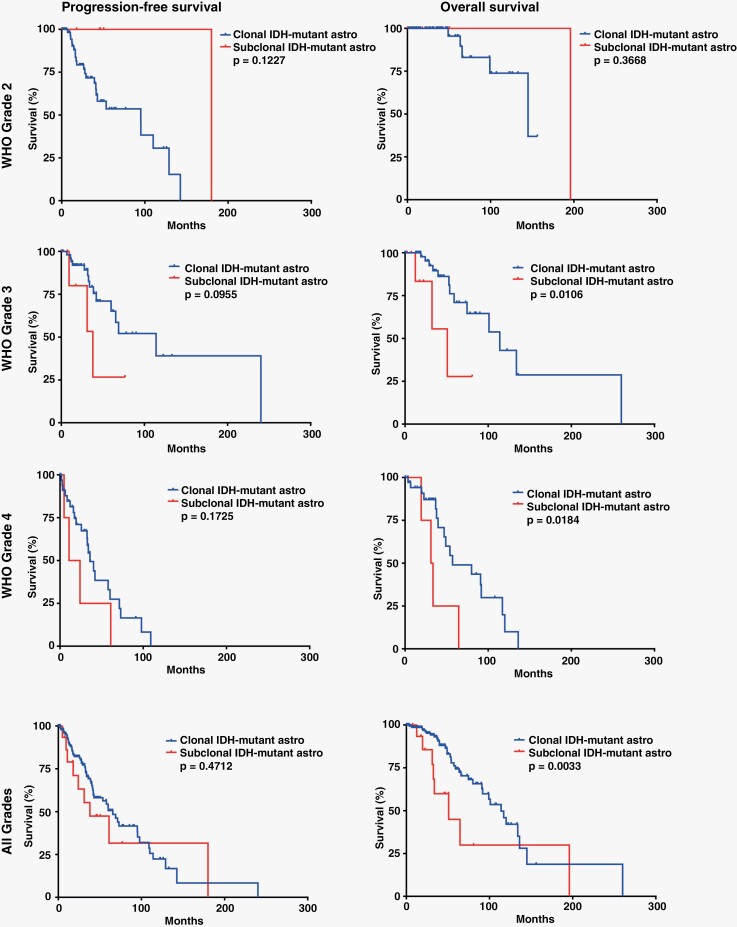
Kaplan–Meier survival curves demonstrating decreased overall survival in WHO grade 3 and 4 subclonal IDH-mutant astrocytomas compared to their clonal IDH-mutant astrocytoma counterparts.

No significant differences were noted in recurrence/progression-free survival between the subclonal and clonal IDH-mutant astrocytomas, although there were nonsignificant trends toward shorter progression-free survival in subclonal grade 3 (38 vs. 114 months; *P* = .0955) and grade 4 (17.4 vs. 36 months; *P* = .1725) astrocytomas compared to grade 3 and 4 clonal IDH-mutant astrocytomas. The subclonal astrocytomas did have significantly shorter overall survival (OS) compared to clonal astrocytomas (50.8 vs. 114 months; *P* = .0033), and this was present in grade 3 (50.8 vs. 114 months; *P* = .0106) and grade 4 (32.4 vs. 57.1 months; *P* = .0184) but not grade 2 (*P* = .3668) cases (Figure 4 and Table 2). Compared to IDH-wild-type glioblastoma from the same publicly available cohorts (*n* = 983); however, subclonal IDH-mutant astrocytomas had significantly better progression-free survival (*P* < .0001) and OS (*P* = .0005) (Supplementary Figure 1).

## Discussion

Our results demonstrate that while rare, subclonal IDH mutation does occur and appears to have a significantly worse effect on survival compared to grade-matched IDH-mutant astrocytomas with clonal IDH mutation. Subclonality of *IDH1* mutations was identified in the institutional cases included in this report via 2 distinct but complimentary methods: The relative percentage of tumor cells with IDH1 R132H immunoreactivity compared to the number of total tumor cells with other pathogenic mutations detectable by immunohistochemistry, including loss of nuclear ATRX reactivity and increased nuclear p53 immunostaining (Figures 1 and 3), and the confirmation of this finding with the ratio of VAF of *IDH1* mutations compared to the VAF of other pathogenic mutations in the same tumor sample to establish an *IDH1* mutation TCF consistent with subclonality. While the institutional cases were insufficient to determine the clinical impact of subclonal IDH mutations, we were able to leverage two large publicly available glioma datasets^7,35^ to evaluate the prognostic impact of this finding. These data suggest that there is a significantly adverse impact on overall survival associated with subclonal *IDH1* mutation compared to grade 3 and grade 4 clonal cohorts (Table 2 and Figure 4), a relationship that has been suggested previously.^24^

Although many studies have demonstrated intercellular genomic heterogeneity as a fundamental feature of diffuse glioma and other solid cancer progressions,^19,20,22,25,35,42^ IDH mutation is considered to be an early oncogenic event initiating glioma evolution,^4,40,43–45^ and as such is generally found with high penetrance across the tumor cell population of a given glioma. The identification of subclonal IDH1 mutation suggests that in a minority of cases, this mutation was either acquired in a subpopulation of an extant IDH-wild-type glioma or the mutation was present early in tumorigenesis and then lost in a subpopulation of tumor cells that had an apparent survival advantage so as to subsequently become the majority of the tumor volume.^23^ Institutional case 1 had an extremely low IDH-mutant TCF, as well as a mutation in the mismatch-repair gene *MSH2* and corresponding elevated tumor mutation burden (14 mutations/Mb), which could provide a potential mechanism for the *IDH1* mosaicism; the genomic instability resulting from this mutation^14^ may have either resulted in *IDH1* mutation in a subpopulation of tumor cells late in tumor progression or a revertant mutation in the *IDH1* R132 codon. Alternatively, increasing CNV with tumor progression/molecular evolution or as a result of radiation and chemotherapies^13,17,31,46^ could result in the loss of the *IDH1* R132H mutated allele in a subset of cells, a possibility in institutional case 2. Given the lack of additional molecular features typically associated with IDH-wild-type glioblastoma in any of the institutional or public subclonal astrocytomas (*EGFR* amplification, simultaneous gain of chromosome 7/loss of chromosome 10, and *TERT* promoter mutation) as well as the DNA methylation profiling result consistent with IDH-mutant astrocytoma in institutional case 1 and all 13 of the publicly available subclonal cases for which DNA methylation profiling classification data were available, it is unlikely that these tumors represent IDH-wild-type glioblastomas with late *IDH1* mutation. Additionally, the presence of a mutation in an MMR-related gene does not appear to be of increased frequency in cases with subclonal *IDH1* mutation, seen in 2/20 total subclonal cases compared to 6/156 clonal cases (*P* = .2264) (Tables 1 and 2). Sublclonal IDH-mutant astrocytomas also have significantly better progression-free survival and OS compared to IDH-wild-type glioblastoma (Supplementary Figure 1), which suggests that they are not simply IDH-wild-type tumors with a late *IDH1* mutation in a relatively small subclone. The presence of subclonal *IDH1* mutation is, therefore, more likely due to *IDH1* mutation as an early event, followed by loss of this mutation or loss of the allele and clonal expansion of the “IDH-wild-type subclone.”^21,40,47^ The likelihood that the IDH mutation is lost in a large subset of cells is of particular interest in light of the methylation profiling results,^39^ as these cases appear to maintain their IDH-mutant methylation phenotype despite the partial loss of the underlying IDH mutation that originally drove this phenotype, suggesting that the epigenetic footprint of IDH mutation may be strong enough to persist even in the absence of the mutation itself.

Previous studies have estimated tumor cell purity and determined the TCF containing specific mutations extrapolated from genomic data using more complicated formulas establishing the expected and observed VAF for each identified mutation.^24,27,48–50^ Here, we opted for a simplified method of estimating the TCF with *IDH1* mutation, ie, comparison of the *IDH1* VAF with the VAF of other known pathogenic mutations present in the same tumor sample (*TP53* or *ATRX*). Given that these additional mutations would not be expected to be present in the non-neoplastic background brain parenchyma, the ratio of *IDH1* VAF to these other mutations allows for estimation of maximum *IDH1* TCF. For example, if the *IDH1* VAF is 0.1 (2 of the 10 cells have a heterozygous *IDH1* mutation) and the *TP53* VAF is 0.4 (8 of the 10 cells are heterozygous for a particular pathogenic *TP53* mutation), the maximum *IDH1* TCF is 0.25, whereas if the hemizygous *TP53* VAF is 0.4 (one allele is deleted and 4 of the 10 cells have a particular pathogenic *TP53* mutation), the maximum *IDH1* TCF is 0.50, and if the *ATRX* VAF is 0.4 in a *male* patient (4 of the 10 cells have a hemizygous pathogenic *ATRX* mutation), the maximum *IDH1* TCF is 0.50. This simplified method also has the benefit of being more clinically useful than the more complex models, as calculations of the *IDH1* TCF can be easily performed from data routinely reported in NGS studies from institutional and reference laboratories. While all of the cases included in this study had *IDH1* R132H mutations, it should be noted that a similar effect may be present in non-canonical *IDH1* or *IDH2* mutations. Subclonality of IDH mutations in these cases would be undetectable by current IHC testing; however, they would be easily identifiable in routine NGS studies.

In conclusion, while the mechanism of this subclonal mosaic *IDH1* mutation remains uncertain, these data demonstrate that diffuse astrocytomas with subclonal *IDH1* mutation may have worse clinical outcomes than their grade-matched clonal *IDH1*-mutant counterparts, although these patients have later recurrences and survive significantly longer than IDH-wild-type glioblastoma patients. This feature is important to recognize as the vast majority of pathology reports simply state a qualitative presence or absence of *IDH1/2* mutation (as well as other pathogenic mutations), even when NGS studies report the relative VAF of each alteration. The possibility of subclonal *IDH1* mutation is especially important to investigate in cases with discordant IHC and NGS findings or methylation results suggesting an IDH mutation in the presence of minimal IDH1 R132H IHC staining, as in institutional case 1. These findings suggest a potentially overlooked source of variability in clinical outcome in cohorts of patients with IDH-mutant astrocytomas, and demonstrate the importance of quantitative IDH mutation assessments. It is important to consider the subclonal tumor pattern and continuing molecular evolution in determining diagnostic criteria, personalizing treatment, and designing effective clinical trials.

## Supplementary Material

vdad069_suppl_Supplementary_Figure_S1

vdad069_suppl_Supplementary_Material

## Data Availability

Portions of the data presented in this manuscript were derived from https://www.cbioportal.org and https://www.cancer.gov/tcga. Additional data is available from the corresponding authors upon request.

## References

[1] Louis DN , Perry A, Wesseling P, et al. The 2021 WHO classification of tumors of the central nervous system: A summary. Neuro Oncol. 2021;23(8):1231–1251. 34185076 10.1093/neuonc/noab106 PMC8328013

[2] Ostrom QT , Price M, Neff C, et al. CBTRUS statistical report: Primary brain and other central nervous system tumors diagnosed in the United States in 2015-2019. Neuro Oncol. 2022;24(suppl 5):v1–v95. 36196752 10.1093/neuonc/noac202 PMC9533228

[3] Parsons DW , Jones S, Zhang X, et al. An integrated genomic analysis of human glioblastoma multiforme. Science. 2008;321(5897):1807–1812. 18772396 10.1126/science.1164382 PMC2820389

[4] Yan H , Parsons DW, Jin G, et al. IDH1 and IDH2 mutations in gliomas. N Engl J Med. 2009;360(8):765–773. 19228619 10.1056/NEJMoa0808710 PMC2820383

[5] Hartmann C , Meyer J, Balss J, et al. Type and frequency of IDH1 and IDH2 mutations are related to astrocytic and oligodendroglial differentiation and age: A study of 1,010 diffuse gliomas. Acta Neuropathol. 2009;118(4):469–474. 19554337 10.1007/s00401-009-0561-9

[6] Brennan CW , Verhaak RG, McKenna A, et al; TCGA Research Network. The somatic genomic landscape of glioblastoma. Cell. 2013;155(2):462–477. 24120142 10.1016/j.cell.2013.09.034 PMC3910500

[7] Ceccarelli M , Barthel FP, Malta TM, et al; TCGA Research Network. Molecular profiling reveals biologically discrete subsets and pathways of progression in diffuse glioma. Cell. 2016;164(3):550–563. 26824661 10.1016/j.cell.2015.12.028 PMC4754110

[8] Cimino PJ , Zager M, McFerrin L, et al. Multidimensional scaling of diffuse gliomas: Application to the 2016 World Health Organization classification system with prognostically relevant molecular subtype discovery. Acta Neuropathol Commun. 2017;5(1):39. 28532485 10.1186/s40478-017-0443-7 PMC5439117

[9] Aoki K , Nakamura H, Suzuki H, et al. Prognostic relevance of genetic alterations in diffuse lower-grade gliomas. Neuro Oncol. 2018;20(1):66–77. 29016839 10.1093/neuonc/nox132 PMC5761527

[10] Shirahata M , Ono T, Stichel D, et al. Novel, improved grading system(s) for IDH-mutant astrocytic gliomas. Acta Neuropathol. 2018;136(1):153–166. 29687258 10.1007/s00401-018-1849-4

[11] Yang RR , Shi ZF, Zhang ZY, et al. IDH mutant lower grade (WHO Grades II/III) astrocytomas can be stratified for risk by CDKN2A, CDK4 and PDGFRA copy number alterations. Brain Pathol. 2020;30(3):541–553. 31733156 10.1111/bpa.12801 PMC8018138

[12] Brat DJ , Aldape K, Colman H, et al. cIMPACT-NOW update 5: Recommended grading criteria and terminologies for IDH-mutant astrocytomas. Acta Neuropathol. 2020;139(3):603–608. 31996992 10.1007/s00401-020-02127-9 PMC8443062

[13] Mirchia K , Sathe AA, Walker JM, et al. Total copy number variation as a prognostic factor in adult astrocytoma subtypes. Acta Neuropathol Commun. 2019;7(1):92. 31177992 10.1186/s40478-019-0746-y PMC6556960

[14] Suwala AK , Stichel D, Schrimpf D, et al. Primary mismatch repair deficient IDH-mutant astrocytoma (PMMRDIA) is a distinct type with a poor prognosis. Acta Neuropathol. 2021;141(1):85–100. 33216206 10.1007/s00401-020-02243-6 PMC7785563

[15] Richardson TE , Snuderl M, Serrano J, et al. Rapid progression to glioblastoma in a subset of IDH-mutated astrocytomas: a genome-wide analysis. J Neurooncol. 2017;133(1):183–192. 28421459 10.1007/s11060-017-2431-y

[16] Wu CC , Jain R, Neto L, et al. MR imaging phenotype correlates with extent of genome-wide copy number abundance in IDH mutant gliomas. Neuroradiology. 2019;61(9):1023–1031. 31134296 10.1007/s00234-019-02219-8 PMC7587301

[17] Richardson TE , Sathe AA, Xing C, et al. Molecular signatures of chromosomal instability correlate with copy number variation patterns and patient outcome in IDH-mutant and IDH-wildtype astrocytomas. J Neuropathol Exp Neurol. 2021;80(4):354–365. 33755138 10.1093/jnen/nlab008

[18] Liu Y , Sathe AA, Abdullah KG, et al. Global DNA methylation profiling reveals chromosomal instability in IDH-mutant astrocytomas. Acta Neuropathol Commun. 2022;10(1):32. 35264242 10.1186/s40478-022-01339-2 PMC8908645

[19] Johnson BE , Mazor T, Hong C, et al. Mutational analysis reveals the origin and therapy-driven evolution of recurrent glioma. Science. 2014;343(6167):189–193. 24336570 10.1126/science.1239947 PMC3998672

[20] Suzuki H , Aoki K, Chiba K, et al. Mutational landscape and clonal architecture in grade II and III gliomas. Nat Genet. 2015;47(5):458–468. 25848751 10.1038/ng.3273

[21] Favero F , McGranahan N, Salm M, et al. Glioblastoma adaptation traced through decline of an IDH1 clonal driver and macro-evolution of a double-minute chromosome. Ann Oncol. 2015;26(5):880–887. 25732040 10.1093/annonc/mdv127 PMC4405282

[22] Bai H , Harmanci AS, Erson-Omay EZ, et al. Integrated genomic characterization of IDH1-mutant glioma malignant progression. Nat Genet. 2016;48(1):59–66. 26618343 10.1038/ng.3457 PMC4829945

[23] Lopez GY , Oberheim Bush NA, Phillips JJ, et al. Diffuse midline gliomas with subclonal H3F3A K27M mutation and mosaic H3.3 K27M mutant protein expression. Acta Neuropathol. 2017;134(6):961–963. 29063183 10.1007/s00401-017-1780-0 PMC6078201

[24] Luo S , Zhu S, Liao J, et al. IDH clonal heterogeneity segregates a subgroup of non-1p/19q codeleted gliomas with unfavourable clinical outcome. Neuropathol Appl Neurobiol. 2021;47(3):394–405. 33098109 10.1111/nan.12671

[25] Nicholson JG , Fine HA. Diffuse glioma heterogeneity and its therapeutic implications. Cancer Discov. 2021;11(3):575–590. 33558264 10.1158/2159-8290.CD-20-1474

[26] Morgan KM , Danish S, Xiong Z. Diffuse astrocytoma with mosaic IDH1-R132H-mutant immuno-phenotype and low subclonal allele frequency. Intractable Rare Dis Res. 2022;11(1):43–45. 35261853 10.5582/irdr.2022.01019 PMC8898389

[27] Bai M , Wang X, Zhang H, et al. Dissecting and analyzing the Subclonal mutations associated with poor prognosis in diffuse glioma. Biomed Res Int. 2022;2022:4919111. 35496054 10.1155/2022/4919111 PMC9039777

[28] Capper D , Jones DTW, Sill M, et al. DNA methylation-based classification of central nervous system tumours. Nature. 2018;555(7697):469–474. 29539639 10.1038/nature26000 PMC6093218

[29] Serrano J , Snuderl M. Whole genome DNA methylation analysis of human glioblastoma using illumina BeadArrays. Methods Mol Biol. 2018;1741:31–51. 29392688 10.1007/978-1-4939-7659-1_2

[30] Huse JT , Snuderl M, Jones DT, et al. Polymorphous low-grade neuroepithelial tumor of the young (PLNTY): An epileptogenic neoplasm with oligodendroglioma-like components, aberrant CD34 expression, and genetic alterations involving the MAP kinase pathway. Acta Neuropathol. 2017;133(3):417–429. 27812792 10.1007/s00401-016-1639-9 PMC5325850

[31] Lyon JF , Vasudevaraja V, Mirchia K, et al. Spatial progression and molecular heterogeneity of IDH-mutant glioblastoma determined by DNA methylation-based mapping. Acta Neuropathol Commun. 2021;9(1):120. 34193272 10.1186/s40478-021-01221-7 PMC8243907

[32] Umphlett M , Bilal KH, Martini ML, et al. IDH-mutant astrocytoma with EGFR amplification-Genomic profiling in four cases and review of literature. Neurooncol Adv. 2022;4(1):vdac067. 35669011 10.1093/noajnl/vdac067 PMC9159664

[33] Cerami E , Gao J, Dogrusoz U, et al. The cBio cancer genomics portal: an open platform for exploring multidimensional cancer genomics data. Cancer Discov. 2012;2(5):401–404. 22588877 10.1158/2159-8290.CD-12-0095 PMC3956037

[34] Gao J , Aksoy BA, Dogrusoz U, et al. Integrative analysis of complex cancer genomics and clinical profiles using the cBioPortal. Sci Signal. 2013;6(269):pl1. 23550210 10.1126/scisignal.2004088 PMC4160307

[35] Jonsson P , Lin AL, Young RJ, et al. Genomic correlates of disease progression and treatment response in prospectively characterized gliomas. Clin Cancer Res. 2019;25(18):5537–5547. 31263031 10.1158/1078-0432.CCR-19-0032 PMC6753053

[36] Richardson TE , Sathe AA, Kanchwala M, et al. Genetic and epigenetic features of rapidly progressing IDH-mutant astrocytomas. J Neuropathol Exp Neurol. 2018;77(7):542–548. 29741737 10.1093/jnen/nly026 PMC6005148

[37] Galbraith K , Kumar A, Abdullah KG, et al. Molecular correlates of long survival in IDH-wildtype glioblastoma cohorts. J Neuropathol Exp Neurol. 2020;79(8):843–854. 32647886 10.1093/jnen/nlaa059

[38] Chkheidze R , Raisanen J, Gagan J, et al. Alterations in the RB Pathway with inactivation of RB1 characterize glioblastomas with a primitive neuronal component. J Neuropathol Exp Neurol. 2021;80(12):1092–1098. 34850045 10.1093/jnen/nlab109

[39] Jamshidi P , McCord M, Galbraith K, et al. Variant allelic frequency of driver mutations predicts success of genomic DNA methylation classification in central nervous system tumors. Acta Neuropathol. 2023;145(3):365–367. 36700952 10.1007/s00401-023-02542-8 PMC11096842

[40] Mazor T , Chesnelong C, Pankov A, et al. Clonal expansion and epigenetic reprogramming following deletion or amplification of mutant IDH1. Proc Natl Acad Sci U S A. 2017;114(40):10743–10748. 28916733 10.1073/pnas.1708914114 PMC5635900

[41] Malta TM , de Souza CF, Sabedot TS, et al. Glioma CpG island methylator phenotype (G-CIMP): Biological and clinical implications. Neuro Oncol. 2018;20(5):608–620. 29036500 10.1093/neuonc/nox183 PMC5892155

[42] Barthel FP , Johnson KC, Varn FS, et al; GLASS Consortium. Longitudinal molecular trajectories of diffuse glioma in adults. Nature. 2019;576(7785):112–120. 31748746 10.1038/s41586-019-1775-1 PMC6897368

[43] Watanabe T , Nobusawa S, Kleihues P, Ohgaki H. IDH1 mutations are early events in the development of astrocytomas and oligodendrogliomas. Am J Pathol. 2009;174(4):1149–1153. 19246647 10.2353/ajpath.2009.080958 PMC2671348

[44] Philip B , Yu DX, Silvis MR, et al. Mutant IDH1 promotes glioma formation in vivo. Cell Rep. 2018;23(5):1553–1564. 29719265 10.1016/j.celrep.2018.03.133 PMC6032974

[45] Turcan S , Rohle D, Goenka A, et al. IDH1 mutation is sufficient to establish the glioma hypermethylator phenotype. Nature. 2012;483(7390):479–483. 22343889 10.1038/nature10866 PMC3351699

[46] Cohen A , Sato M, Aldape K, et al. DNA copy number analysis of Grade II-III and Grade IV gliomas reveals differences in molecular ontogeny including chromothripsis associated with IDH mutation status. Acta Neuropathol Commun. 2015(1);3:34. 26091668 10.1186/s40478-015-0213-3 PMC4474351

[47] Luchman HA , Chesnelong C, Cairncross JG, Weiss S. Spontaneous loss of heterozygosity leading to homozygous R132H in a patient-derived IDH1 mutant cell line. Neuro Oncol. 2013;15(8):979–980. 23757293 10.1093/neuonc/not064 PMC3714156

[48] Aran D , Sirota M, Butte AJ. Systematic pan-cancer analysis of tumour purity. Nat Commun. 2015;6:8971. 26634437 10.1038/ncomms9971 PMC4671203

[49] Landau DA , Carter SL, Stojanov P, et al. Evolution and impact of subclonal mutations in chronic lymphocytic leukemia. Cell. 2013;152(4):714–726. 23415222 10.1016/j.cell.2013.01.019 PMC3575604

[50] Tarabichi M , Salcedo A, Deshwar AG, et al. A practical guide to cancer subclonal reconstruction from DNA sequencing. Nat Methods. 2021;18(2):144–155. 33398189 10.1038/s41592-020-01013-2 PMC7867630

