# Supplementary figures and images for: The prognostic impact of subclonal IDH1 mutation in grade 2–4 astrocytomas
Source: Neurooncol Adv. 2023 May 29;5(1):vdad069. doi: 10.1093/noajnl/vdad069 (PMC10263115; doi:10.1093/noajnl/vdad069)

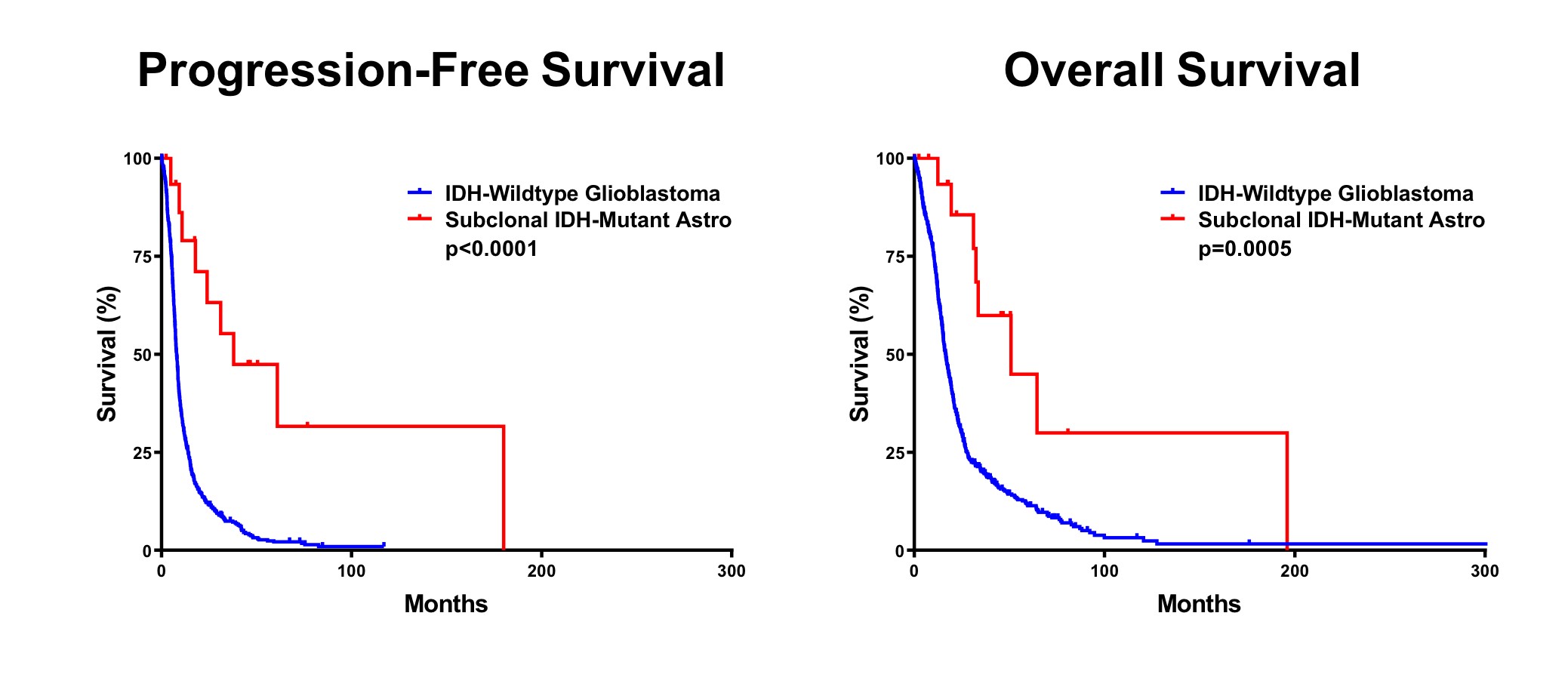

Supplement: vdad069_suppl_Supplementary_Figure_S1 [file vdad069_suppl_supplementary_figure_s1.jpeg]
