# Supplementary material for: The prognostic impact of subclonal IDH1 mutation in grade 2–4 astrocytomas
Source: Neurooncol Adv. 2023 May 29;5(1):vdad069. doi: 10.1093/noajnl/vdad069 (PMC10263115; doi:10.1093/noajnl/vdad069)
Supplement: vdad069_suppl_Supplementary_Material [file vdad069_suppl_supplementary_material.docx]

**Supplemental Figure 1.** Kaplan-Meier survival curves demonstrating survival differences between subclonal IDH-mutant astrocytomas and IDH-wildtype glioblastoma.
